# Public beliefs and willingness to accept COVID-19 vaccines among adults in South-Western Nigeria: A cross-sectional study

**DOI:** 10.3934/publichealth.2023001

**Published:** 2023-01-17

**Authors:** Itse Olaoye, Aniebet Ekong, Abiona Samuel, Eirini Kelaiditi, Kyriaki Myrissa, Tsemaye Jacdonmi, Famokun Gboyega

**Affiliations:** 1 World Health Organization Ondo State Field Office, Akure, Nigeria; 2 Faculty of Sport, Allied Health and Performance Science, St Mary's University, Twickenham, London, UK, TW1 4SX; 3 Faculty of Health and Social Sciences, Bournemouth University, Bournemouth; 4 Ondo State Primary Health Care Development Agency, Akure, Nigeria; 5 Ondo State Ministry of Health, Akure, Nigeria

**Keywords:** COVID-19, vaccines, beliefs, willingness, vaccination hesitancy, Nigeria

## Abstract

**Background:**

Despite the government's and development partners' unmatched efforts to ensure that every eligible person receives vaccinations, there have been concerns about vaccine fear, government mistrust, vaccine hesitancy and rejection expressed by the public, as well as various conspiracy theories involving the COVID-19 vaccines. This study assessed the public beliefs and willingness to accept COVID-19 vaccines and related factors among residents of Ondo State, Nigeria.

**Methods:**

Using a convenience sample technique, a cross-sectional survey of the adult population was carried out in the months of February and March of 2022. Factors influencing beliefs and willingness to accept COVID-19 vaccines were found by using univariate and multivariate statistical analysis.

**Results:**

306 out of 323 respondents completed the survey and were included in the final analysis. The respondents mean age was 28.16 ± 16.2 years. Although *n* = 223, 72.9% of respondents reported to have received at least one dose of COVID-19 vaccines, (*n* = 205) 67.0% believed COVID-19 vaccines to be effective. Among the individuals who had not yet had any COVID-19 vaccinations, 2.6% (*n* = 8) of respondents were willing to accept the vaccines, whereas 14.1% (*n* = 43) were unwilling. Respondents' beliefs about the efficacy of COVID-19 vaccines were influenced by their gender, occupation, religion and educational attainment (p < 0.005).

**Conclusion:**

The study revealed a good level of positive beliefs about the vaccine, which was mirrored in vaccination history. However, those who had not yet received the vaccine were unwilling to do so, opening the door for more aggressive risk communication to be able to alter the course of events. In addition to addressing additional COVID-19 vaccination myths, we advise policy-makers to develop communication strategies that emphasise the safety of the COVID-19 vaccine. It is advised that all relevant stakeholders be included in government COVID-19 vaccination programmes by sharing timely, transparent information that fosters accountability.

## 1. Introduction

The novel coronavirus disease 2019 (COVID-19), which was first discovered in Wuhan, China in December 2019, was described as a global public health pandemic by the World Health Organization (WHO) on the 11^th^ of March 2020 [1]. Since the onset of the COVID-19 pandemic, the disease has spread to over 215 countries [2]. Globally, the pandemic has continued to pose a threat to socioeconomic stability, food security, trade, health systems, education systems and infrastructure in both high- and low-income countries. As of the 1^st^ of July 2022, there have been 545,226,550 confirmed cases of COVID-19, including 6,334,728 deaths reported to the WHO [3]. In Ondo State, Nigeria, there have been 104,396 cases tested for COVID-19, 5173 confirmed cases, 109 deaths, 4749 cases discharged and 315 cases on admission as of the 1^st^ of July 2022 [4].

COVID-19 vaccines were introduced to combat the ongoing COVID-19 pandemic. On 8 December 2020, the first COVID-19 vaccination was administered outside of a clinical research environment [5]. By 8 December 2021, 55.9% of the global population was estimated to have received at least one dose of a COVID-19 vaccine [6]. Only 57% of nations, almost all of which are high-income nations, had vaccinated 70% of their total population as of May 2022, as compared to almost a billion individuals in low-income nations who are still unvaccinated [3]. In March 2021, Nigeria received about 4 million doses of AstraZeneca/Oxford vaccines through the COVID-19 Vaccines Global Access facility, i.e., a partnership between the Coalition for Epidemic Preparedness Innovations, Global Alliance for Vaccines and Immunizations (GAVI), United Nation Children's Fund (UNICEF) and WHO [7]. Priority groups such as frontline healthcare workers, security personnel, strategic leaders and other public personnel identified as eligible for the first phase of the COVID-19 vaccination were first vaccinated in March 2021, as coordinated by the National Primary Health Care Development Agency, through the State's Primary Health Care Development Agency, and in partnership with development partners [8],[9]. However, there has been implementation of other phases of the vaccination programme: Phase 2 (16 August 2021 to 16 November 2021) and Phase 3 (17 November 2021 to date), giving wider access to other COVID-19 vaccines such as Moderna and Pfizer to the general public [9].

As of the 1^st^ of July 2022, 20.8% of the total eligible population have been fully vaccinated with the COVID-19 vaccine, and only 10.7% of the population have been partially vaccinated with one dose of COVID-19 vaccine [9]. Specifically, in Ondo State, only 9.5% of the target population have been fully vaccinated against COVID-19, and 16% partially vaccinated [9]. A high vaccination coverage is typically required for the immunisation campaign to be successful. However, despite the government's and development partners' unmatched efforts to ensure that every eligible person receives vaccinations, there have been concerns about vaccine fear, government mistrust, vaccine hesitancy and rejection expressed by the public, as well as various conspiracy theories surrounding the origin of COVID-19 and the speculated motives behind the vaccines in Nigeria [10].

There have been few studies in Nigeria among health workers and the general public that assessed the level of awareness, perceptions and willingness to accept COVID-19 vaccines [11]–[13]. Adedeji-Adenola and colleagues [13], who explored the factors influencing COVID-19 vaccine uptake among adults in Nigeria, found sociodemographic factors such as occupation, religion and education to be predictors of COVID-19 vaccination awareness. They also found health workers to have more of a positive perception towards COVID-19 vaccination than non-health workers. A study [12] conducted prior to the introduction of COVID-19 vaccines in Nigeria found that older age, males, trust in government, trust in public health authorities and confidence in vaccine developers were significantly associated with COVID-19 vaccine acceptance. A similar study [11] was conducted among health workers in Ondo State, Nigeria prior to the availability of the COVID-19 vaccine in the country and state [11]. They found that only 53.5% of the health workers had positive perceptions of the COVID-19 vaccine, and only slightly more than half (55.5%) were willing to receive vaccination. Predictors of willingness to receive the COVID-19 vaccine included having a positive perception of the vaccine and a higher perceived risk of contracting COVID-19.

There has been no study in Ondo State evaluating public's perceptions, beliefs and willingness to receive COVID-19 vaccines post-introduction of the vaccines in the state.

Nigeria is a country that is multi-ethnic, multi-cultural and multi-religious [14]. Experiences from the government's expanded immunisation programme indicate that different parts of the country have varying levels of vaccination coverage [15]. When compared to the northern states, higher vaccination coverage rates have been seen in the southern states [16]. Additionally, intra-state coverages reveal that urban areas have higher coverage than rural areas. Inequalities in socioeconomic status and literacy levels are also present [16]. Given these facts, it is clear that it is important to understand the context-specific variables that can affect the uptake of COVID-19 vaccines in Ondo State, South-West Nigeria. Moreover, the low vaccination rates in Ondo State necessitate an understanding of the beliefs and willingness to accept the COVID-19 vaccine. Findings from this study will be very useful in guiding the ongoing implementation of the vaccination programme and the subsequent phases of the programme. Therefore, this study aimed to assess the beliefs and willingness to receive COVID-19 vaccines among the public in Ondo State, Nigeria.

## 2. Materials and methods

### 2.1. Study setting

**Figure 1.**
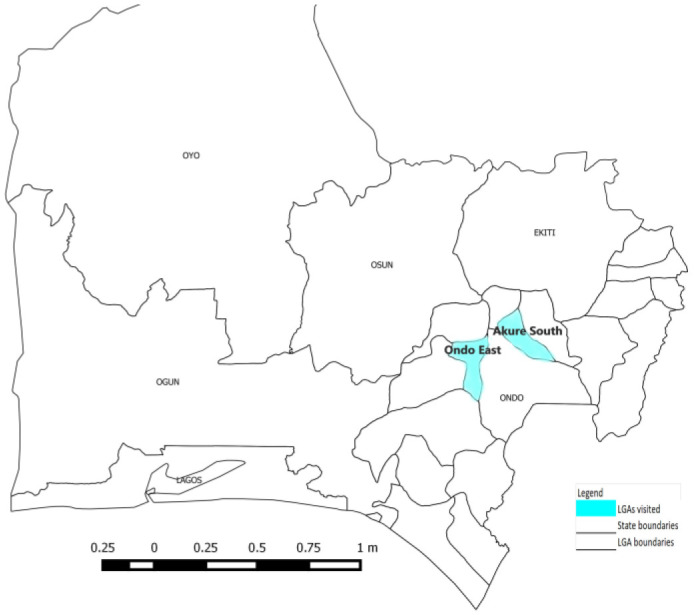
Map of Ondo State, Nigeria showing selected LGAs.

The study area was Ondo State in the southwestern part of Nigeria. It has a land area of approximately 14,789 km^2^ and a projected population of 5.3 million people, as based on the 2006 national census population in Nigeria, which projected a growth rate of 3.0% [17]. The state is divided into 18 local government areas (LGAs) and 203 political wards. This study was conducted in two LGAs in the state, i.e., Akure South, which is considered an urban LGA, and Ondo East, a rural LGA (Figure 1). The Akure South LGA is the headquarters of Ondo State. It has 11 wards and a total population of 610,727 as the projected population. The Ondo East LGA has 10 wards and a projected total population of 129,262.

### 2.2. Study design, study participants and sampling

This was a cross-sectional study conducted from February to March 2022 in the Akure and Ondo East LGAs of Ondo State. Selection of the LGAs was based on convenience sampling. Study participants were Nigerian residents who were 18 years or older living in Ondo state. A sample size of 323 was estimated by applying an online sample size calculator [18] to a total population of 5,361,003 for Ondo State at a 95% confidence interval, a 5% margin of error and an 80% response rate.

### 2.3. Data collection and analysis

A questionnaire was designed and incorporated into the Google survey form and interview-administered to respondents by trained research assistants. The study questionnaire contained questions on sociodemographic information such as age, gender, occupation, religion, level of education, history of COVID-19 testing and history of COVID-19 vaccination. The belief in COVID-19 vaccines and participants' willingness to receive the vaccine was assessed by using single-item questions, each with “yes”, “no” and “maybe” answer options and multiple-choice follow-up responses for reasons that were established based on previous literature [19]. The study questionnaire was piloted among 10% of the intended sample size in communities outside of the LGAs selected for the study. Pretesting was carried out to ensure internal consistency and eliminate ambiguities. Test-retest reliability for single items was established by using intraclass correlation, which was 0.8. Face and content validity was done by the main author and the researchers.

All completed questionnaires were analysed by using IBM's Statistical Package for the Social Sciences (SPSS) version 27. Descriptive statistics were computed to generate frequencies, means and standard deviations. Chi-square analysis was performed to explore associations between participants' sociodemographic characteristics and beliefs on COVID-19 vaccines and vaccination history. A significance level of p < 0.05 was considered statistically significant.

### 2.4. Ethical consideration

Ethical approval was sought and granted by the Health Research and Ethics committee of the Ondo State Ministry of Health with protocol number OSHREC 22/03/2022/429. Written consent was obtained from all participants before partaking in the study. Confidentiality was assured and the data collected were anonymized.

## 3. Results

A total of 306 of 323 respondents completed the survey forms and were included in the final statistical analysis of this study, giving a response rate of 94.7%.

### 3.1. Sociodemographic characteristics of respondents

Sixty percent (*n* = 185) of the responders were females, while 39.5% (*n* = 121) were males. About 50.7% (*n* = 155) of the respondents were in the 34–49 age range. Only 1.6% (*n* = 5) of the population was over 65 years (Table 1). The average age was 28.16 ± 16.2 years. The participants worked in a range of occupations: 15.4% (*n* = 47) were students, 22% (*n* = 69) were civil/public servants and 39.2% (*n* = 120) were involved in commerce or trading. Health professionals made up 9% (*n* = 28), retired employees made up 4.9% (*n* = 14) and unemployed respondents made up 9.5% (*n* = 28). Fewer respondents (*n* = 13) had no education, as compared to 60.5% (*n* = 185) with tertiary education, 29.4% (*n* = 90) with secondary education and 4.3% (*n* = 13) with no education. The majority of respondents (91.8%; *n* = 281) identified as Christian (Table 1).

**Table 1.** Respondents' sociodemographic characteristics.

Characteristics	Frequency	Percent
**Age group**		
18 to 33 years	110	35.9
34 to 49 years	155	50.7
50 to 65 years	36	11.8
>65 years	5	1.6
Mean age ± SD	28 ± 16.2	
**Gender**		
Male	121	39.5
Female	185	60.5
**Occupation**		
Business/trader	120	39.2
Civil/public servant	69	22.5
Health worker	28	9.2
Housewife	15	4.9
Retired	14	4.6
Student	47	15.4
Unemployed	13	4.2
**Religion**		
Christian	281	91.8
Islam	25	8.2
**Education**		
None	13	4.2
Primary	18	5.9
Secondary	90	29.4
Tertiary	185	60.5

### 3.2. Respondent's beliefs and willingness regarding COVID-19 vaccines

Most respondents 67.0% (*n* = 205) believed in the effectiveness of COVID-19 vaccines. Twenty-one percent (*n* = 65) were indifferent on the effectiveness of COVID-19 vaccines, while 11.8% (*n* = 36) did not believe in the vaccine effectiveness (Table 2). There were more respondents 72.9% (*n* = 223) who had received at least one dose of COVID-19 vaccine than those who had not 27.1% (*n* = 83). Among those who had not received a single dose of any COVID-19 vaccine, we explored their willingness to be vaccinated. A very low proportion of respondents (2.6%; *n* = 8) indicated their willingness to receive COVID-19 vaccines; others (14.1%; *n* = 43) were not willing to take the vaccines, while 10.5% (*n* = 32) were hesitant on taking the COVID-19 vaccine. Regarding the reasons for the unwillingness or indecision expressed by respondents' beliefs about the vaccines, the majority (53.3%; *n* = 40) expressed concerns about the side effects and safety of the COVID-19 vaccines, 26.7% (*n* = 20) were not sure of its efficacy and 12.0% (*n* = 9) did not trust the vaccination programme and also did not believe in the existence of the COVID-19 virus. Other reasons included the fact that the vaccines were produced outside of Nigeria (*n* = 3; 4%) and religious beliefs (*n* = 4; 5.3%) (Table 2).

**Table 2.** Respondents' beliefs, willingness, COVID-19 vaccination status and COVID-19 test history.

Characteristics	Frequency	Percent
**Belief in COVID-19 vaccine**		
Yes	205	67
No	36	11.8
Maybe	65	21.2
**COVID-19 vaccination history (at least one dose)**		
Yes	223	72.9
No	83	27.1
**Willingness to take COVID-19 vaccine (*n* = 83)**		
Yes	8	2.6
No	43	14.1
Maybe	32	10.5
Reasons for non-willingness to take COVID-19 vaccine (*n* = 75)**		
COVID-19 does not exist	9	12.0
COVID-19 is going away	1	1.3
I am not sure of its efficacy	20	26.7
The vaccine was made outside of Nigeria	3	4.0
I do not trust the vaccination programme	9	12.0
I am too young and do not need it	6	8.0
I am worried about its side effects and safety	40	53.3
It is against my religion	4	5.3
No reason	1	1.3
Others	5	6.7
**History of COVID-19 testing**		
Yes	33	10.8
No	273	89.2

*Note: ** multiple responses allowed.

### 3.3. Association between sociodemographic characteristics and belief in COVID-19 vaccines

Sociodemographic factors such as respondents' age group, gender, occupation, religion and level of education were all statistically significant with belief in COVID-19 vaccines (p < 0.005). Respondents' history of COVID-19 vaccination and testing were also statistically significant with belief in COVID-19 vaccines (p < 0.005) (Table 3). Respondents between the age of 34 to 49 years more likely to believe in COVID-19 vaccine efficacy than those older than 65 years. A higher proportion of males believed in the vaccine effectiveness than females. As anticipated, health workers had better perception, whereas respondents who were unemployed had poor perceptions about the vaccines. Christians had better perceptions about the COVID-19 vaccines than Muslims. Those with no or only primary school education had poor perceptions than those with a tertiary education (Table 3). Respondents without a history of COVID-19 testing and vaccination had poor perceptions about COVID-19 vaccines than those who had previously been tested for COVID-19 and had received at least one dose of a COVID-19 vaccine.

**Table 3.** Association between respondents' characteristics and beliefs about COVID-19 vaccines.

Characteristics	Belief in COVID-19 vaccine			Chi-square (X^2^)	df	p-value
	Yes *n* = 205 (%)	No *n* = 26 (%)	Maybe *n* = 65 (%)			
**Age group**				19.948	6	0.003*
18 to 33 years	69(62.7)	11(10.0)	30(27.3)			
34 to 49 years	112(72.3)	14(9.0)	29(18.7)			
50 to 65 years	22(61.1)	8(22.2)	6(16.7)			
>65 years	2(40.0)	3(60.0)	0(0.0)			
**Gender**				7.899	2	0.019*
Male	90 (74.4)	7(5.8)	24(19.8)			
Female	115(62.2)	29(15.7)	41(22.2)			
**Occupation**				74.451	12	<0.001*
Business/trader	69(57.5)	19(15.8)	32(26.7)			
Civil/public servant	60(87.0)	4(5.8)	5(7.2)			
Health worker	28(100)	0(0)	0(0)			
Housewife	2(13.3)	4(26.7)	9(60.0)			
Retired	12(85.7)	2(14.3)	0(0.0)			
Student	32(68.1)	5(10.6)	10(21.3)			
Unemployed	2(15.4)	2(15.4)	9(69.2)			
**Religion**				15.585	2	<0.001*
Christian	197(70.1)	29(10.3)	55(19.6)			
Islam	8(32.0)	7(28.0)	10(40.0)			
**Level of education**				114.017	6	<0.001*
None	1(7.7)	7(53.8)	5(38.5)			
Primary	3(16.7)	8(44.4)	7(38.9)			
Secondary	39(43.3)	15(16.7)	36(40.0)			
Tertiary	162(87.6)	6(3.2)	17(9.2)			
**History of COVID-19 testing**				12.374	2	0.002*
Yes	31(93.9)	0(0.0)	2(6.1)			
no	174(63.7)	36(13.2)	63(23.1)			
**COVID-19 vaccination history**				155.283	2	<0.001*
Yes	194(86.6)	4(1.8)	26(11.6)			
no	11(13.4)	32(39)	39(47.6)			

*Note: degree of freedom (df), *Significance level (P) ≤ 0.05.

### 3.4. Association between sociodemographic characteristics and COVID-19 vaccination history

Sociodemographic factors, which included age group, occupation, religion and level of education were significantly associated with COVID-19 vaccination history (p < 0.005). Only gender was not significantly associated with COVID-19 vaccination history (Table 4). Similar to the belief in COVID-19 vaccines, those who were between 34 to 49 years old were more likely to be vaccinated, while those who were above 65 years were less likely to be vaccinated. Health workers were more likely to receive COVID-19 vaccines than those who were unemployed. In the same vein, Christians and those with a tertiary level of education showed a higher likelihood of being vaccinated than those who were Muslims and with no education, respectively. History of COVID-19 testing was also significantly associated with COVID-19 vaccination history (p < 0.005). Those who had previously been tested for COVID-19 were more likely to receive a COVID-19 vaccination (Table 4).

**Table 4.** Association between respondents' characteristics and COVID-19 vaccination history.

Characteristics	COVID-19 vaccination history		Chi-square (X^2^)	df	p-value
	Yes *n* = 224 (%)	No *n* = 82 (%)			
**Age group**			15.675	3	0.001*
18 to 33 years	70(63.6)	40(36.4)			
34 to 49 years	128(82.6)	27(17.4)			
50 to 65 years	24(66.7)	12(33.3)			
>65 years	2(40.0)	3(60.0)			
**Gender**			0.410	1	0.522
Male	91(75.2)	30(24.8)			
Female	133(71.9)	52(28.1)			
**Occupation**			65.934	6	<0.001*
Business/trader	83(69.2)	37(30.8)			
Civil/public servant	62(89.9)	7(10.1)			
Health worker	28(100.0)	0(0.0)			
Housewife	3(20.0)	12(80.0)			
Retired	12(85.7)	2(14.3)			
Student	34(72.3)	13(27.7)			
Unemployed	2(15.4)	11(84.6)			
**Religion**			19.208	1	<0.001*
Christian	215(76.5)	66(23.5)			
Islam	9(36.0)	16(64.0)			
**Level of education**			104.612	3	<0.001*
None	1(7.7)	12(92.3)			
Primary	1(5.6)	17(94.4)			
Secondary	55(61.1)	35(38.9)			
Tertiary	167(90.3)	18(9.7)			
**History of COVID-19 testing**			10.651	1	0.001*
Yes	32(97.0)	1(3.0)			
No	192(70.3)	81(29.7)			

*Note: degree of freedom (df), *Significance level (P) ≤ 0.05.

## 4. Discussion

The aim of the present study was to investigate the beliefs of the public and willingness of adults to accept COVID-19 vaccination in Ondo State, South-West Nigeria. Findings from this study revealed two-thirds of the respondents to have a positive belief in the effectiveness of COVID-19 vaccines. This agrees with a previous study of COVID-19 vaccine acceptance in Nigeria that also found an acceptance level of two-thirds [20], but lower than the findings by Olomofe and colleagues [21], who looked into predictors of COVID-19 uptake. Belief in a health intervention has been found to correlate with ensuing health behaviour and acceptance of interventions [22],[23]. Interestingly, the percentage of respondents who reported to have had at least one dose of a COVID-19 vaccine exceeded the percentage of those who believed in the effectiveness of COVID-19 vaccines by 5.9%. Given that a person's perception that a vaccine is beneficial is reported to influence vaccination action from the health-belief model of illness behaviour [23],[24], this disparity may imply that there were individuals who claimed indifference to—or expressed disbelief in the effectiveness of COVID-19 vaccines—but got vaccinated nonetheless. Moreover, the population who reported indifference to the effectiveness of COVID-19 vaccines was larger in number than those who were indecisive on taking a COVID-19 vaccine. Conversely, respondents who did not believe in the effectiveness of COVID-19 vaccines were fewer in number than those who responded that they were not willing to get a COVID-19 vaccine. These findings further suggest that expressed beliefs/attitudes sometimes differ from actual intentions and subsequent actions [25]. The intention-behaviour gap, which defines the inability to turn intentions into action, is also consistent with this study's findings. Although people wish to engage in recommended behaviour(s), many fail to do so [26].

Additionally, for those yet to receive any dose of a COVID-19 vaccine, safety issues remain a top concern, as more than half of the respondents expressed their unwillingness to be vaccinated due to safety reasons. This may not be surprising considering the various myths and misconceptions surrounding the development and deployment of COVID-19 vaccines during the early days of the pandemic—most of which still linger [24],[27]–[29]. Several authors also found that safety was a top concern about the willingness to be vaccinated with COVID-19 vaccines [23],[24],[27],[29]. Likewise, more than a quarter of respondents (26%) were not sure of the efficacy of COVID-19 vaccines. Similarly, 12% of the respondents expressed distrust with government vaccination programmes on COVID-19. This lack of confidence in the efficacy of the vaccine may be explained by the misconceptions that the development of COVID-19 vaccines was rushed, and that proper checks and balances were not done [30]–[32]. Transparency and prompt information are crucial for promoting beliefs since they can positively influence trust. The lack of trust in a government vaccination programme to deliver COVID-19 vaccination could be linked to the persistent belief by some people that COVID-19 does not exist and may just be a hoax to siphon money by the government and its agents. These efficacy and trust issues have also been reported by other researchers regarding their studies on the acceptance of COVID-19 vaccines [28],[29],[33]. Governments should aim to win the confidence and trust of the populace. We can approach this problem by using the lessons we have learned from the past belief-induced misrepresentation of poliomyelitis immunisation in Nigeria. Political and religious divisions, a lack of community involvement and widespread suspicion of government intentions and those of the international community were all contributing factors to the disruption of the vaccination campaign, which was designed to benefit the public [34].

Our study found a respondent's age, gender, occupation, religion, educational level to be factors that influence individual beliefs regarding the effectiveness of COVID-19 vaccines, and this is also reflected in their vaccination history; individuals who had gotten the COVID-19 vaccine were more likely to have a positive belief about the vaccines. This is congruent with findings from similar studies that showed that these variables positively correlate with the likelihood of getting vaccinated with a COVID-19 vaccine [13],[29]. However, we found that young adults (34 to 49 years old) were more likely to be vaccinated than older ones. This refutes findings from previous authors who reported that much older people were more likely to be vaccinated with a COVID-19 vaccine [20],[35]. Though more males (75.2%) than females (71.9%) had been vaccinated, the association between gender and the belief in the effectiveness of COVID-19 vaccines differed from vaccination history, as there was no association between the gender and vaccination statuses of respondents. This differs from the findings by other authors, who found that the male sex positively correlates with COVID-19 vaccination [13],[21]. According to COVID-19 epidemiological statistics [3],[4],[12], men are more impacted than women. However, these findings may be because multiple factors relating to the individuals may have been responsible for the association in the first instance.

The present study's findings on how religion affects people's willingness to get COVID-19 vaccines is not entirely surprising. This was similar to how the poliomyelitis vaccine was received in Nigeria [34]. Nigeria is renowned for being a strongly religious nation with respect for religious authorities who shape attitudes and beliefs [36]. The importance of religious leaders' direct participation in the spread of accurate information about vaccines, as well as in the resolution of difficulties relating to non-compliance with COVID-19 vaccination, is highlighted by their extremely strong influence. Education level was a significant factor in beliefs regarding the COVID-19 vaccine and the history of vaccination. People and geographic areas in Nigeria with low literacy rates have been reported to have mistrust or negative beliefs regarding vaccines [34].

In our study, we discovered that respondents' occupation had an impact on their immunisation and beliefs regarding the COVID-19 vaccine. People who were gainfully employed showed more positive beliefs about COVID-19 vaccines. As anticipated, every healthcare professional in this study had received at least one dose of the COVID-19 vaccination and displayed more favourable beliefs about the vaccine. This result is consistent with research from Ethiopia and Nigeria [13],[37]. Due to their frequent contact with sick people, health workers are at a high risk of contracting COVID-19; hence, they should continue to be prioritized for COVID-19 vaccinations. They are crucial to the immunisation programme's success as well. According to Qattan and colleagues [38] their knowledge and views determine their recommendation to other non-health professionals, so all cadres of health workers should actively participate in assuring health education.

## 5. Study limitations implications for practice

This study adds a better understanding to the beliefs and willingness to receive COVID-19 vaccines among the public in Ondo State of South-West Nigeria, and it allows us to compare practices between regions of the country. It also provides current scenarios of public beliefs and willingness with regards to COVID-19 vaccination.

Our study revealed a disparity between what people profess and their actual action. This gives room for risk communication to be able to change the course of action, and this should be aggressively explored by policy-makers. Additionally, policy-makers should design communication activities that speak to the safety of COVID-19 vaccines, while not neglecting other aspects of misconceptions around COVID-19 vaccines. Strategies targeted at using males to influence their female relatives to get vaccinated should be actively encouraged, as demonstrated in the lower level of belief and vaccination history of females in this study's findings. While this study showed that gender influenced belief in vaccine effectiveness and not actual vaccination status, there is a good relationship that indicates that people follow a course of health action in which they believe [22],[24]. Besides, concerns of females around the effects of vaccines on their future reproductive capability [34] must be addressed through special women-only community fora and discussion sessions. Policy-makers should also engage with stakeholders by sharing transparent, timely and financial information when needed, especially with community leaders, to promote accountability and trust in government COVID-19 vaccination programmes.

## 6. Study limitations

This research has several limitations. First of all, the convenient sampling method employed to select the study site is inherently biased [39], and our study was limited to two LGAs; as a result, it might not be representative of the population of Ondo State. Future studies with a larger sample size and more participants with lower literacy levels are needed to allow for more analysis and understanding of these demographic factors. Second, respondents self-reported their COVID-19 vaccination history; interviewers did not look for COVID-19 vaccination cards. As a result, social desirability biases may have led to an overreporting of the study's findings regarding vaccination history. Third, we do not assert a causal relationship between sociodemographic characteristics and COVID-19 beliefs and vaccination history due to the cross-sectional design of the study. Despite the aforementioned drawbacks, this study is a novel attempt to close a gap in the literature on COVID-19 vaccine beliefs and can be used to inform future COVID-19 planning and policy in Ondo State, Nigeria. To the best of our knowledge, this is the first study to inquire about the acceptability of COVID-19 vaccines among the Ondo people.
